# Transactions of the Virginia Society of Surgeon Dentists

**Published:** 1844-12

**Authors:** S. Lethbridge, James D. McCabe

**Affiliations:** President.; Secretary.


### ARTICLE V.

*Transactions of the Virginia Society of Surgeon Dentists.* At their Second Annual Meeting, held in the town of Petersburg, October 9th, 1844.

The President, Dr. S. Lethbridge, took the chair at 12, M.

The Secretary called over the names of members, and read the excuses of absentees.

The proceedings of last meeting were read.

A vacancy occurring in the examining committee, in consequence of the absence of some of its members, the President appointed Drs. John G. Wayt, A. Pierce and L. Murrell to fill said vacancy.

The President announced the meeting duly organized, and ready to proceed to business.

John H. Blankenship presented an application for examination and membership. Referred to examining committee.

On motion, the society adjourned until this evening, at 7 o'clock.

*Evening Session.*

The society assembled, pursuant to adjournment, at 7 o'clock, P. M.

The President called to order.

Dr. J. D. McCabe announced to the society the death of professor H. H. Hayden, with appropriate remarks. Whereupon, the following preamble and resolutions were adopted:

"The Virginia Society of Surgeon Dentists have heard with deep and heartfelt grief the death of H. H. Hayden, M. D., D. D. S., Professor of Dental Physiology in the Baltimore College of Dentistry, President of the American Society of Surgeon Dentists, and an *Honorary Member of this Society;* one, whose contributions to the cause of science, generally, and whose indefatigable labors for the improvement and elevation of dental science particularly, have won for him imperishable fame, and justly entitled him to the gratitude of the profession, and the approbation of the world:

*Be it therefore Resolved,* That in common with his friends, and the dental profession, we deplore the loss we have mutually sustained in the death of our distinguished professional brother, professor H. H. Hayden, and we do hereby tender to his family our most affectionate sympathies in this their heavy bereavement.

*Resolved,* That as a mark of respect to the memory of the deceased, the members of this society will wear the usual badge of mourning for thirty days.

*Resolved,* That the Secretary be directed to forward a copy of these resolutions to the family of Dr. Hayden, and to the faculty of the Baltimore College of Dentistry.

The committee appointed to procure an act of incorporation from the general assembly of Virginia, made a verbal report: Whereupon, it was

*Ordered,* That the committee be continued, with instructions to renew the application to the next legislature, and to address to each member of both houses a circular setting forth the objects and wishes of this society.

On motion the President was added to the committee.

The examining committee reported the name of John H. Blankenship, an applicant for admission, who had presented a *Thesis* "On Plugging the Teeth," and had been rigidly examined on the theory and practice of dental surgery, and that they unanimously recommend him to the society as qualified for membership and the attendant honors.

Admitted by unanimous ballot.

The Treasurer presented his report, which was received, and ordered to be filed with the papers of the society.

On motion, the society proceeded to election of officers for the ensuing year, when the following gentlemen were duly chosen:

Drs. S. LETHBRIDGE, *President.*
JOHN GEO. WAYT, *Vice-President.*
JAMES D. McCABE, *Cor. and Rec. Secretary.*
S. M. SHEPHERD, ———————,
JOHN McCONNELL,
W. W. H. THACKSTON,
L. MURRELL,
A. W. PIERCE.
T. B. HAMLINE,
} *Executive, Examining and Pub. Committee.*

A letter was received from Dr. W. W. H. Thackston, stating that circumstances "of domestic character, and of paramount importance," interposed to prevent his attendance, and delivery of the annual address which he had prepared. Whereupon,

*Ordered,* That he be requested to publish the address in the American Journal of Dental Science.

Dr. John Geo. Wayt was elected to deliver the annual address on the second Wednesday in October, 1845.

Dr. J. D. McCabe read an essay on "*Diseases of First Dentition.*" Dr. J. G. Wayt, an essay on "*Dental Irritation.*" Dr. S. M. Shepherd exhibited *models* of a case of "dental irregularity," representing the several stages of cure, and explained his mode of treatment. Dr. S. Lethbridge exhibited several remarkable teeth extracted by himself. Dr. Wayt gave a verbal description of a case of diseased antrum, treated successfully by him.

Dr. McCabe gave a verbal description of a case of disease of *parotid glands*, occasioned by *cutting* the inferior *dens sapientiæ*, and which was relieved by their removal, after a continuance of twelve or eighteen months.

Several interesting verbal reports of cases were made.

The following members were appointed to prepare essays to be read before the society at its next annual meeting: Drs. S. Lethbridge, John Geo. Wayt, S. M. Shepherd, J. D. McCabe, L. Murrell, A. W. Pierce, John H. Blankenship.

On motion of Dr. McConnell,

*Resolved,* That the members of this society be requested to furnish written reports to the next and succeeding annual meetings of all anomalous and interesting cases, occurring in their practice during each year.

On motion,

*Resolved,* That Drs. McCabe and Wayt be requested to furnish copies of their papers presented to this meeting for publication in the American Journal of Dental Science.

On motion of Dr. McCabe,

*Resolved, unanimously,* That as a token of their high esteem for the professional character and personal merit of William Tatam, M. D., of Norfolk, Va., as well as in acknowledgment for his services in their behalf in the last general assembly of Virginia, this society do hereby create him an honorary member, and that he be furnished with a diploma without the usual fee.

On motion,

*Resolved*, That candidates for examination before the committee of this society, shall be required to present a written *thesis* on some subject, selected by themselves, unless they, for good reasons, be excused by the committee.

On motion,

*Ordered*, That the next meeting of this society be held in the city of Richmond on the 2d Wednesday in October, 1845.

On motion,

The society then adjourned to the 2d Wednesday in October, 1845.

S. LETHBRIDGE, D. D. S., *President*.

James D. McCabe, D. D. S., *Secretary*.

